# Neuro-Behavioral Status and the Hippocampal Expression of Metabolic Associated Genes in Wild-Type Rat Following a Ketogenic Diet

**DOI:** 10.3389/fneur.2019.00065

**Published:** 2019-02-05

**Authors:** Ya Ling, Dan-dan Wang, Yu-xiao Sun, Dong-jing Zhao, Hong Ni

**Affiliations:** ^1^Division of Brain Science, Institute of Pediatric Research, Children's Hospital of Soochow University, Suzhou, China; ^2^The First Affiliated Hospital of Wenzhou Medical University, Wenzhou, China

**Keywords:** ketogenic diet, zinc transporter, ApoE, cPLA2, ANXN7, Apoa1, Pdk4, wild-type rat

## Abstract

While a ketogenic diet (KD) is a well-established therapy for medically intractable epilepsy, clinical evidence of relevant adverse events of a KD has also been reported. We asked whether this kind of diet would have deleterious effects on wild-type brain function by evaluating KD-induced biochemical changes in the hippocampus as well as neurobehavioral changes occurring in wild-type rats. Fifty-four Sprague-Dawley rats were randomly assigned to three groups on postnatal day 28 (P28): wild-type rats fed with a KD qd (daily for 4 weeks, KD) or qod (every other day for 4 weeks, KOD), and wild-type rats fed with standard normal laboratory diet (ND). Neurobehavioral changes were observed on P35, P42, and P49. The hippocampal mossy fiber sprouting, the expression levels of zinc transporters (ZnTs) and lipid metabolism related genes were detected by Timm staining, RT-qPCR and western blot analysis, respectively, on P58. The KD-treated KOD and KD groups showed a significant delay of negative geotaxis reflex on P35, but not on P42 or P49. In the open field test, daily KD treatment only led to a reduction in exploratory activity and increased grooming times but induced no significant changes in the scores of vertical activity or delay time. KD qod treated rats (KOD) displayed a slight delay in the place navigation test on P35 compared with the KD group. There were no significant differences in Timm staining among the three groups. In parallel with these changes, KD treatment (both KD and KOD) induced significantly downregulated mRNA levels of Apoa1, Pdk4, and upregulated expression of ApoE, ANXN7, and cPLA2 in the hippocampus when compared with the ND group (except in the case of ApoE in the KOD group). Notably, both the mRNA and protein levels of cPLA2 in the KOD rats were significantly downregulated compared with the KD group but still markedly higher than in the ND group. No significant difference was found in ZnTs among the three groups. Our data suggest that early-life KD can provoke minor neurobehavioral effects in particular a delay in negative geotaxis reflex and an increase in grooming activity. The hippocampal lipid metabolism signaling pathway, especially cPLA2, may be the target of the protective effect of KD on long-term brain injury after developmental seizures.

## Introduction

Ketogenic diet (KD) is a valuable therapy for medically intractable epilepsy that has been applied for nearly 100 years. The KD has recently been experimentally utilized in many neurological disease, including autism spectrum disorders and ACTH-resistant West syndrome (1, 2). Interestingly, a KD can also effectively prevent exercise-induced oxidative stress of Taekwondo athletes (3). On the other hand, it has been reported that a KD can result in a variety of complications, including protein-losing enteropathy, arterial stiffness, nephrolithiasis, cholelithiasis, declined linear growth status, trace mineral deficiencies, and an increased tendency for mood problems (4–9), which limits its usefulness.

Animal data also indicates that a KD is associated with long-term adverse effects of metabolic abnormal diseases such as biotin deficiency, dyslipidemia, signs of hepatic steatosis, etc., (10, 11). However, previous studies have mainly observed the effect of the KD on the function of peripheral organs, while the study of brain function has been less frequent. One animal study using KD-exposed young-adult CD-1 mice showed that KD-prenatal exposure influenced their offspring's neuroanatomy and behavior (12). Another study demonstrated that a gestational KD altered not only maternal metabolic status but also offspring's growth and brain development (13). In humans, Shiohama et al. reported that a ketogenic diet at 4 months of age induced white matter lesions during KD therapy (14). The adverse effects of KD on the cognitive, behavioral, psychosocial regulation and quality of life of school-age children and adolescents were investigated by Lambrechts et al. The results showed an increase in emotional problems (9).

Based on the above evidence, and combined with the encouraging laboratory data that therapeutic diets, including KD, had a beneficial effect on neurophysiological and clinical parameters (although there is controversy about the real clinical effect of therapeutic diets in patients with autism) (15), a more comprehensive study of the neurotoxicological repertoire, particularly of the effects of the KD on brain tissue, is necessary. The purpose of this study is to conduct an investigation of the effect of a high-fat, low-carbohydrate ketogenic diet on neurobehavioral parameters of wild-type young rats.

Energy metabolism signals in the hippocampus have been investigated to understand the anticonvulsant effect of KDs (15). In particular, we have recently shown modulatory effects of the KD on the gene expression of zinc/lipid signal-related genes following recurrent neonatal seizures (16, 17). Ueda et al. previously showed temporarily upregulated expression of lipid metabolism-associated genes in hippocampus, including Apoa1, Gh, Mc4r, Oprk1, and Pdk4, in the sub-chronic phase in a rat model of posttraumatic epilepsy (18). To further clarify the causal relationship of zinc and lipid metabolism signals, KD-induced biochemical changes in the hippocampus, particularly the expression of zinc and lipid transporter related genes occurring in wild-type animals, were compared in this study. The rats were fed with KD starting at postnatal day 28 (1 week after weaning) for the next 30 days.

## Materials and Methods

### Animal Preparation

Sprague-Dawley rats (*n* = 54) with balanced sex at postnatal day 8 (P8) were obtained from the Chinese Academy of Sciences, Shanghai Experimental Animal Center, China. The rats were kept in an environmental control room away from strong light and noise under a 12 h/12 h light/dark cycle. The study was approved by the Institutional Animal Care and Use Committee of the Children's Hospital of Soochow University. The program was approved by the Medical Ethics Committee of Soochow University Children's Hospital. At weaning day P21, the animals were randomly divided into three groups (*n* = 18/group): wild-type rats fed with a KD qd (daily, KD) or qod (every other day, KOD), and wild-type rats fed with standard normal laboratory diet (ND). At P28, rats in the KD and KOD groups received a KD, while rats in ND group received normal diet. The KD formula was reported in detail before (16). The KD (70% fat, 20% protein and no carbohydrate) and normal diet (50% carbohydrate, 20% protein and 4.5% fat) were purchased from the Chinese Academy of Sciences, Shanghai Experimental Animal Center, China.

### Measurement of Blood β-Hydroxybutyrate (BHB) Level

Ketone bodies in blood plasma, especially BHB, is the clinical hallmark of success for the KD. To ensure ketosis, BHB levels were measured with a Keto-detector meter (Beijing Yicheng Bioelectronics Technology Co., Ltd., China) using blood samples obtained via tail vein. Levels of BHB were determined at P35 and P49 following dietary therapy.

### Neurobehavioral Tests

Neurological behavioral parameters of brain injury, including negative geotaxis reflex, plane righting reflex, cliff avoidance reflex and forelimb suspension test, were tested on P35, P42, and P49 according to the procedure previously described (19, 20).

### Open Field Test

The open-field test was performed on P42 and P49 as described previously (21, 22). The number of grids that the rats traversed after they started running (based on the four claws entering the grid), recorded as a horizontal score, the number of hind legs upright (including the front paws climbing the wall or vacated), recorded as a vertical score. The horizontal score is added to the vertical score as the score of locomotor activity. The delay time (time sitting in the central square) and grooming time was also recorded.

### Timm Staining

Because one of our goals is to study the effect of KD on zinc transporter expressions in the wild-type hippocampus and the expression of zinc transporters involved in the pathogenesis of hippocampal mossy fiber sprouting, we also examined the effect of KD on the mossy fiber sprouting of hippocampus in wild-type rats in this study. On P58, some rats were stained with Timm staining (*n* = 6/group). The regenerative sprouting of mossy fibers in the CA3 region and the inner molecular layer of the dentate gyrus of hippocampus were analyzed using a semi-quantitative method (23).

### RT-qPCR

Six randomly selected rats from each group were anesthetized with chloropent (3 ml/kg, i.p.) on P58. The method has been described in detail previously (22). The primers and probes of the 12 genes were designed against GenBank-published sequences with the software Primer Express 2.0 (Applied Biosystems), and the sequences are listed in Table 1. In order to determine the fold change in expression, the 2^−ΔΔ*Cq*^ method of relative quantification was used. Firstly, to determine the qPCR threshold cycle (Cq) of the target mRNAs and the internal control β-actin. Secondly, to calculate the ratios of target genes: β-actin as follows: Target gene: β-actin = 2^Cq(target)−Cq(β−*actin*)^ (ΔCq = Cq target–Cq β-actin). The fold change in expression was then obtained (2^−Δ*CT*^ –method) (24).

**Table 1:** Oligonucleotide primers for RT-qPCR analysis.

**Gene**	**Genbank accession number**	**Primer sequence**
ApoE	NM_001270681.1	F: 5′-ACCTAATGGAGAAGATACA-3′ R: 5′-GAGAATCTTTATTAAGCAAGG-3′ Probe: 5′-FAM-AACTCCATTGCCTCCACCAC-TAMRA-3′
Apoa1	NM_012738	F: 5′-CGATCAGATGCGCGAGAAC-3′ R: 5′-TACTCGATCAGGGTAGGGTGGTT-3′ Probe: 5′-FAM-CCCAGCGCCTGACCGAGATCAA-TAMRA-3′
PDK4	NM_053551	F: 5′-GCTCACACAAGTCAATGGAAAATT-3′ R: 5′-ATGTGGTGAAGGTGTGAAGGAA-3′ Probe: 5′-FAM-CCAGGCCAACCAATCCACATCGTG-TAMRA-3′
Annexin A7	NM_130416	F: 5′-TTGTGGATGTCGTGTCTA-3′ R: 5′-GGCATCATAGTATGTAGGAG-3′ probe: 5′-FAM-CGTTCCAATGACCAGAGGCA-TAMRA-3′
cPLA2	NM_133551	F: 5′-AGATCCTTATCAGCACAT-3′ R: 5′-CACAGGGTTTATATCATTATTG-3′ probe: 5′-FAM-TTGTTCGCTTCCTGCTGTCA-TAMRA-3′
ZnT-1	NM_022853	F: 5′- CGTTGTTGTGAATGCCTTGGT-3′ R: 5′-GGGTTCACACAAAAGTCGTCTTC-3′ probe:: 5′-FAM-TTCTACTTTTCCTGGAAGGGTTGTA-TAMRAM-3′
ZnT-3	NM_001013243	F: 5′- TGGGCGCTGACGCTTACT-3′ R: 5′- GTCAGCCGTGGAGTCAATAGC-3′ probe:: 5′-FAM-ACCACGTTGCCTCCGCACACCT-TAMRAM-3′
ZnT-4	NM_172066.1	F: 5′- GCTGAAGCAGAGGAAGGTGAA−3′ R: 5′- TCTCCGATCATGAAAAGCAAGTAG−3′ probe:5′-FAM-CAGGCTGACCATCGCTGCCGT-TAMRA−3′
ZnT-5	NM_001106404.1	F: 5′- CCAGCACATGTCTGGCCTAA−3′ R: 5′- TTTGCAGTACTTCATGGATTCCA−3′ probe: 5′-FAM-CACTGGCTTCCACGATGTCCTGGCTAT– TAMRA-3′
ZnT-6	NM_001106708.1	F: 5′- CGGCATTATCCCAGGACTCA−3′ R: 5′-CCAGCAAGATCGATCAGAACAA−3′ probe: 5′-FAM- TTCTTGCCCCGCATGAACCCG -TAMRA-3′
ZnT-7	XM_001073594.1	F: 5′- TTGGGATCCGCGTCTGA−3′ R: 5′- CCCTCTAGAAGTGACTCGGTATGG−3′ probe: 5′-FAM-TCGTCTCTGCTGTCACTGCCGCC–TAMRA- 3′

### Western Blot Analysis

Western blot method was used to detect protein levels on P58 (22) (*n* = 6/each group). After blocking in a TBS-T solution, the membrane blots were incubated with rabbit anti-cPLA2 polyclonal antibody (1:800, Eno Gene) overnight at 4°C. Then the blot was incubated with the secondary antibody for 2 h at ambient temperature. Six different blot bands (from six different animals) for each protein were calculated quantitatively using SigmaScan Pro 5. The data were normalized with respect to ratios of β-actin detected on the same blot to control for possible variation in protein loading across samples. Statistical analysis was carried out by *post hoc* comparisons using a Bonferroni test after ANOVA.

### Statistical Analysis

The Timm staining, the mRNA and the protein levels were analyzed with *post hoc* comparisons using a Bonferroni test after ANOVA, depending on the normality of the distribution (Shapiro-Wilk test). The behavioral parameters were analyzed by non-parametric Kruskal-Wallis test using SAS 8.0 statistical software, when statistically significant differences were found, a *post-hoc* Dunn test was used to compare the groups. Data are presented as the mean ± SD, and statistical significance was considered as a *P* < 0.05.

## Results

### Blood BHB Levels

**Figure 1:**
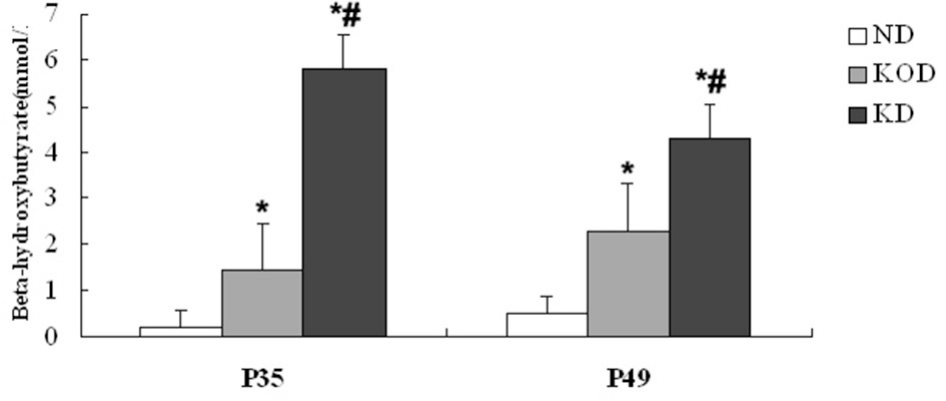
Influence of the KD on blood BHB levels at P35 and P49. ^*^P < 0.001 vs. the ND group;^#^*P* < 0.01 vs. the KOD group.

As shown in Figure 1, at P35 and P49 following dietary treatment, BHB levels in KD-fed rats (KOD: P35: 1.43 ± 0.37 mM, P49: 2.31 ± 0.72 mM; KD: P35: 5.8 ± 0.44 mM, P49: 4.3 ± 1.09 mM) were significantly (*P* < 0.001) higher than those seen in ND-fed animals (ND: P35: 0.2 ± 0.08 mM, P49: 0.5 ± 0.08 mM). In addition, BHB levels in KD group were significantly (*P* < 0.01) higher than those seen in KOD animals. In addition, as shown in Supplementary Figure 1, at P49 following dietary treatment, the weight in KD-fed rats were significantly (*P* < 0.05) lower than those seen in ND-fed animals; meanwhile, weight levels in ND and KOD groups were not significantly different.

### Neurological Behavior

**Figure 2:**
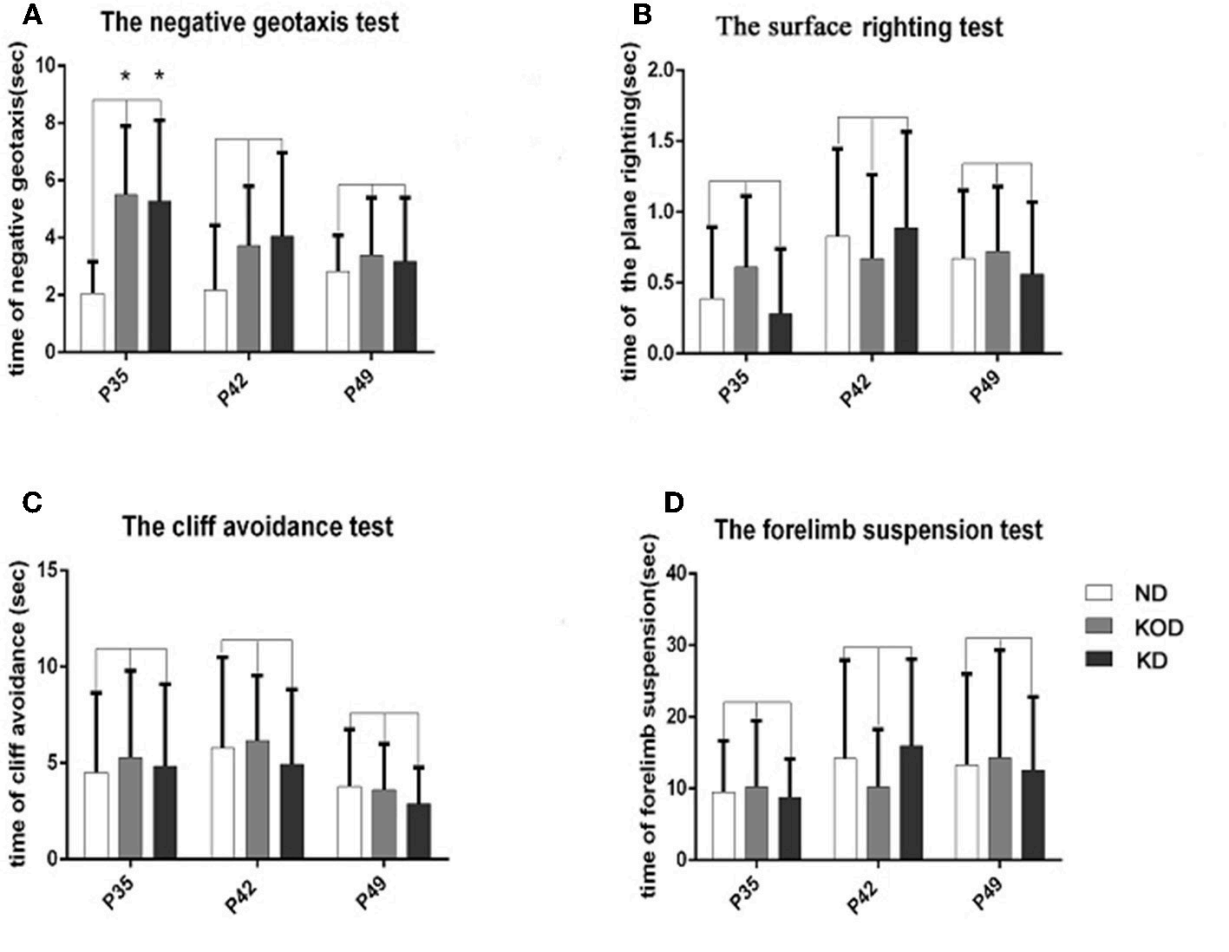
The differences of neurobehavioral development in control and rat pups fed a KD. **(A)** The negative geotaxis reflex in control and rat pups fed with a KD. ^*^P < 0.05 vs. the ND group. **(B)** The surface righting reflex in control and rat pups fed with a KD. *P* > 0.05 vs. the ND group. **(C)** The cliff avoidance reflex in control and rat pups fed with a KD. *P* > 0.05 vs. the ND group. **(D)** The forelimb suspension test in control and rat pups fed with a KD. *P* > 0.05 vs. the ND group. All the data were analyzed by non-parametric Kruskal–Wallis test (*n* = 18/group).

As shown in Figure 2, the KD-treated KOD and KD groups showed a significant delay of negative geotaxis reflex on P35 only (*P* < 0.05, Figure 2A). No other differences were observed in the surface righting reflex, cliff avoidance reflex or forelimb suspension test (Figures 2B–D).

### Open Field Test

**Figure 3:**
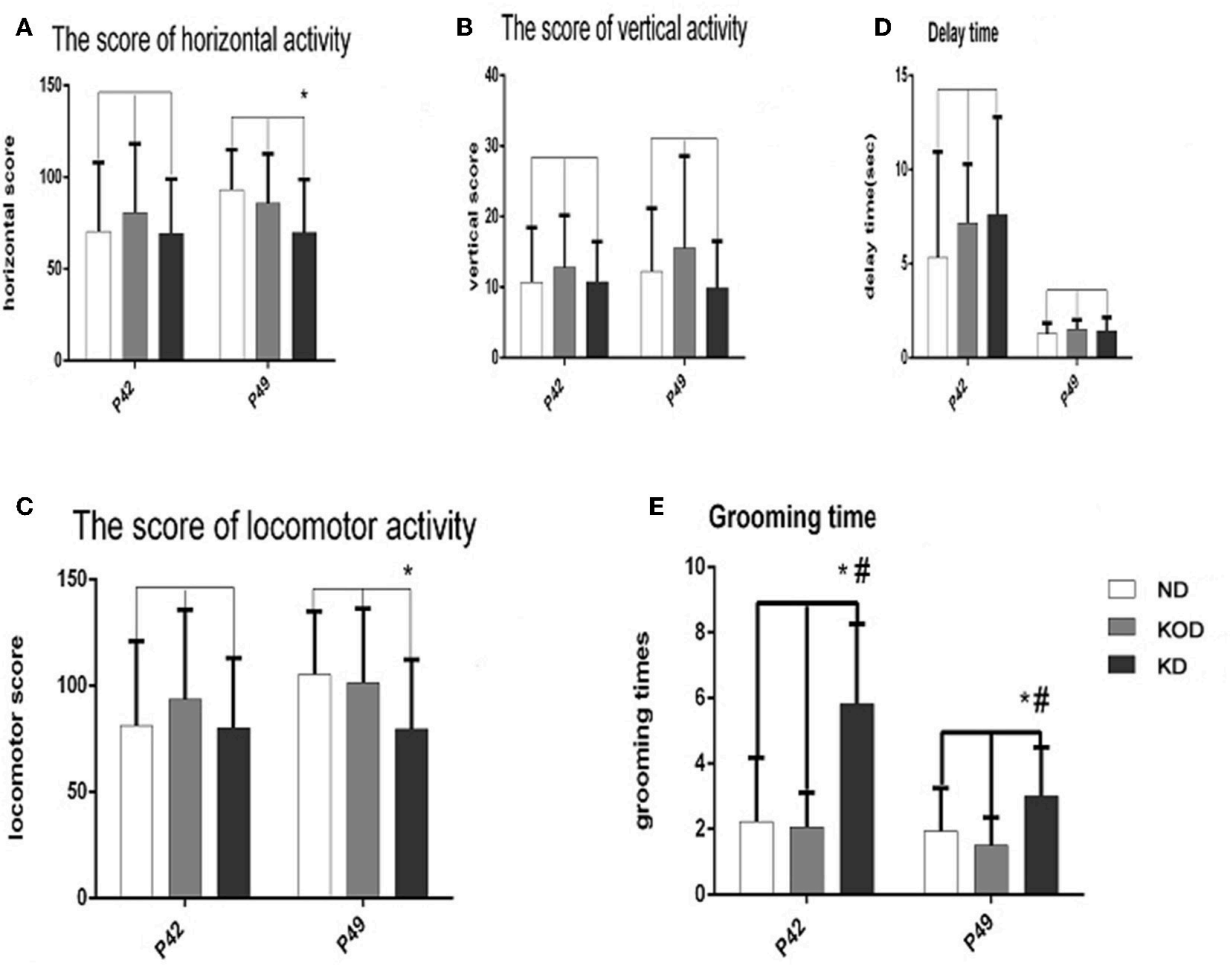
Behavior in the open field. Exploratory activity is reflected by the score of horizontal activity **(A)**, vertical activity **(B)** and the sum of them (locomotor activity) **(C)**. Anxiety-like behavior is reflected by delay time **(D)** and grooming times **(E)**. ^*^P < 0.05, compared with ND,^#^*P* < 0.05, compared with KOD (*n* = 18/group).

The open field test evaluates activity in a novel environment, as well as anxiety and exploration. As shown in Figure 3, administration of a KD did not induce any changes in vertical activity or delay time. However, a reduction in exploratory activity was observed, as shown by the reduced score of horizontal activity and locomotor activity in the daily KD-treated animals (KD group) when compared with the control. In addition, animals of the KD group showed anxiety-like behavior, as exhibited by increased grooming times compared with the other two groups.

### Timm Staining

**Figure 4:**
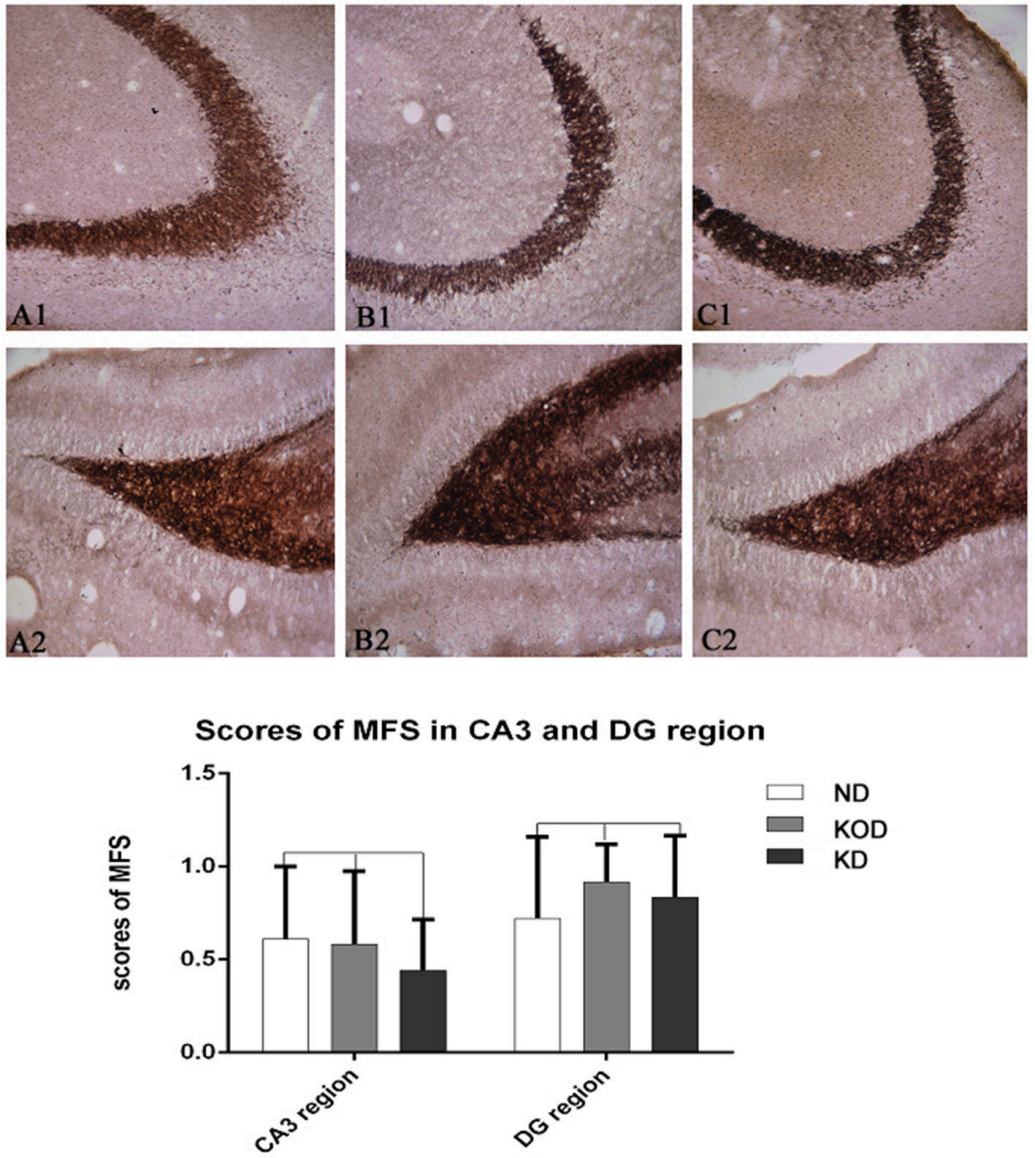
Example of mossy fiber sprouting by Timm staining in ND **(A1,A2)** and KOD **(B1,B2)**, KD **(C1,C2)**. **(A1–C1)** represent the CA3 subfield and **(A2–C2)** represent the dentate gyrus subfield from ND to KD. The Timm staining patterns in the region of stratum pyramidale of CA3 subfield and supragranular of dentate gyrus are not significantly different among the three groups (*n* = 6/group). Calibration bars = 50 μm.

As shown in Figure 4, the Timm staining patterns in the region of stratum pyramidale of the CA3 subfield (Figures 4A1–C1) and supragranular of dentate gyrus (Figures 4A2–C2) are not obviously different among the ND, KOD, and KD groups.

### RT-qPCR Analysis

**Figure 5:**
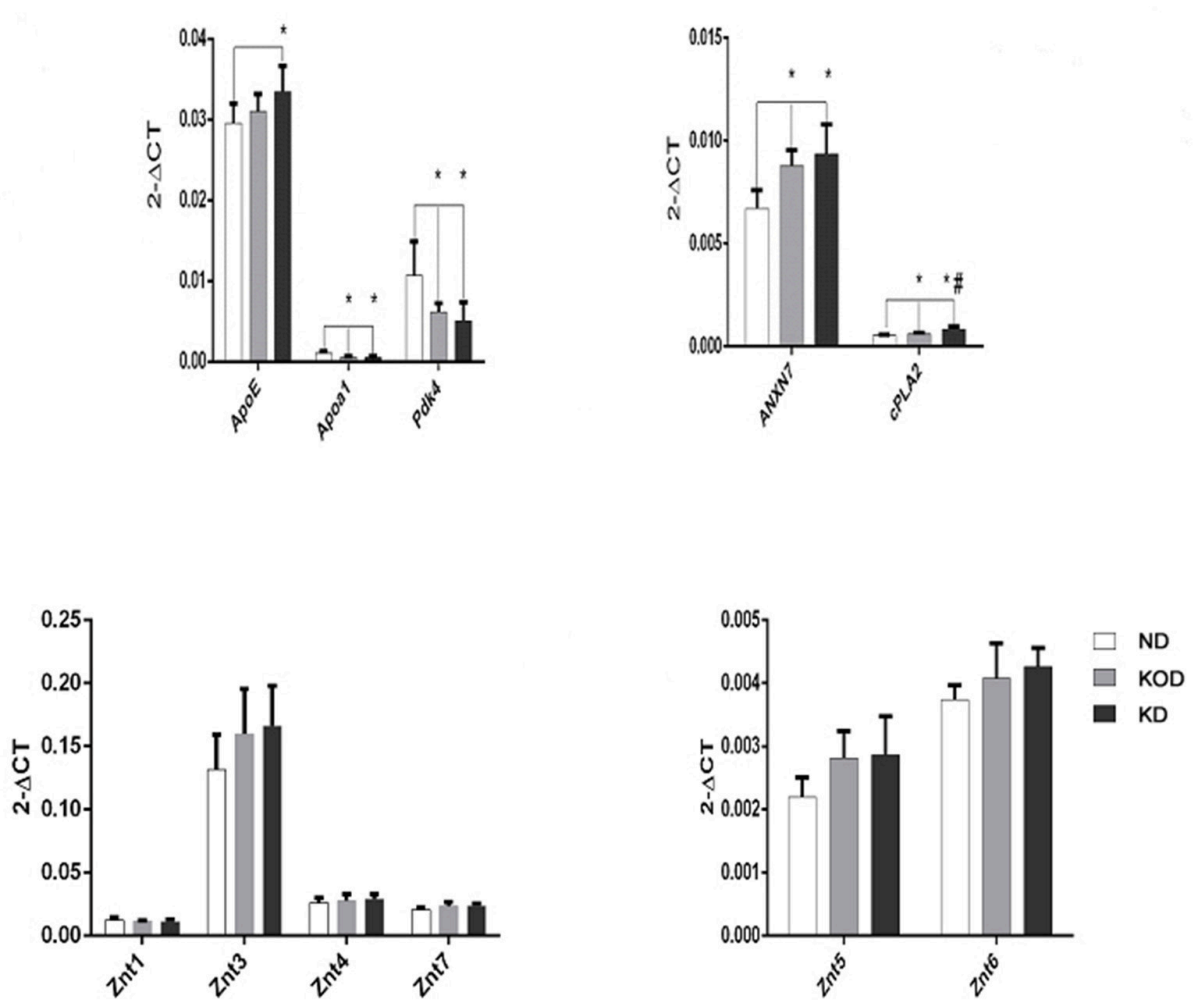
Real-time RT-PCR analysis of hippocampal lipid metabolism and zinc transporter-related genes. ^*^P < 0.05, compared with ND,^#^*P* < 0.05, compared with KOD (*n* = 6/group).

Real time RT-PCR was employed to evaluate the relative gene expression for apolipoprotein E (ApoE), Apoa1(apolipoprotein A-I), Pdk4 (dehydrogenase kinase, isozyme 4), annexin 7 (ANX7), and Ca2+ -dependent phospholipase A2 (cPLA2), which are involved in the regulation of lipid metabolism, and ZnT-1 (zinc transporter 1) and ZnT-3~ZnT-7, which are involved in regulation of zinc metabolism. As shown in Figure 5, among the 11 total genes, 4 lipid metabolism-related genes were downregulated (Apoa1, Pdk4) or upregulated (ANXN7, cPLA2) in the KD-treated KD and KOD groups compared with the ND group. In addition, the mRNA level of ApoE in the KD group was increased significantly compared with the ND group. Further, daily KD-treated rats (KD) showed a significant upregulation of mRNA expression of cPLA2 in the hippocampus when compared with KOD rats. No significant differences were observed in ZnTs expressions among the three groups.

### Western Blot Analysis

**Figure 6:**
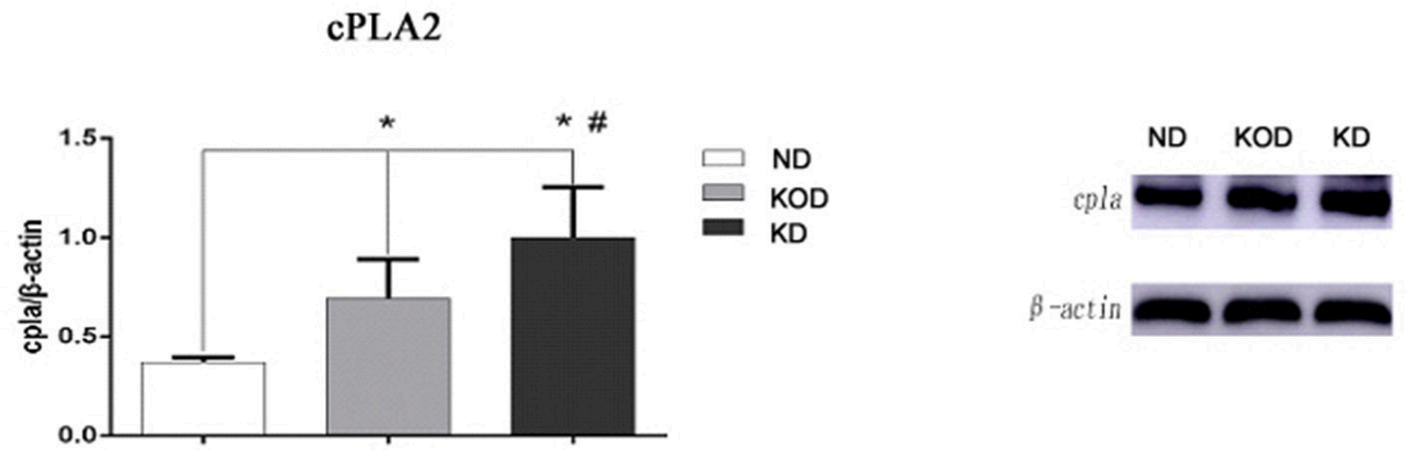
Representative blot of cPLA2 in the hippocampus. Hippocampal extracts from the three groups were separa ted on SDS-PAGE, and protein levels were detected using western blot analysis. Bar represents mean ± SE from six animals in each group. ^*^P < 0.05, compared with ND,^#^*P* < 0.05, compared with KOD (*n* = 6/group).

To validate the RT-PCR results, western blot analysis was performed to evaluate the relative protein levels of cPLA2 in hippocampus. Consistent with the RT-PCR results, KD-treated rats (KOD and KD) had more cPLA2 in the hippocampus when compared with ND rats, while there was higher level of cPLA2 in KD than in KOD (*p* < 0.05). Representative blot is shown on Figure 6.

## Discussion

In this study, we studied the effects of KD administered daily or every other day for 30 days, on behavior and brain expression of metabolism-related genes in healthy wild-type young rats. In a previous study, we have characterized the neurobehavioral effects of the KD following neonatal seizure-induced brain damage, where we have shown the improved neurobehavioral parameters induced by a KD in epileptic rats (16). However, in the two control groups (ND vs. KD), there were no significant differences in the place righting test, cliff avoidance test or open field test. This is partly in line with our current results. Here we show no obvious differences among three groups in the surface righting test, cliff avoidance test, forelimb suspension test, negative geotaxis test (P42, P49), vertical activity or delay time in the open field test. However, in the present study, we found significant differences between the ND and KD groups in the locomotor/horizontal activity and grooming times in the open field test on P49, as well as the negative geotaxis test on P35. This may be explained by the increased BHB levels following dietary treatment. A recent study by Ryan et al found that the low carbohydrate and high fat ketogenic diet (KD) in male rats and mice resulted in an increase in hypothalamic-pituitary-adrenal (HPA) axis tone (25). Thus, we speculate that the increased activation of HPA axis associated with an absence of carbohydrates and increased levels of BHB may be associated with the observed behavioral changes in current study. These new findings are in accordance with some experimental studies. For example, Brownlow et al showed that a KD-induced nutrient ketosis affects behavior in Sprague-Dawley rats in a control and chronic stress environment (26). The adverse effects of KD on cognition in weanling rats has also been reported (27). Chwiej et al. further demonstrated that a KD could induce three-dimensional structural changes in proteins in the DG hippocampal area (28). It was found that rats fed KD showed an increase in social exploration, while there was no difference in activity or anxiety-related behavior or working memory between animals fed KD or standard rodent food (29). Taken as a whole, the current data suggest that early life daily KD treatment in wild-type rats may have no long-term adverse effects on most neurobehavioral parameters, but minor neurobehavioral damage may exist, which merits further investigation.

Research on the effects of KD on wild-type brain gene expression is limited. It has been reported that 8 weeks of KD treatment reduced the levels of BDNF in the striatum of young Wistar rats (30). Three week-KD fed adult mice exhibited increased dopaminergic activity in the motor and somatosensory cortex regions (31). Simeone TA demonstrated that 10- to 14-day *in vivo* KD treatment altered the pathologic sharp waves (SPWs) and high-frequency oscillation complexes generated by the hippocampal CA3 region of epileptic Kv1.1α knockout (KO) mice. The KD also reduced the excitability of moss fibers and increased the paired pulse ratio of moss fiber-CA3 (32). However, The effect of chronic KD treatment on wild-type hippocampal-related gene expression has not been thoroughly investigated. Recently, we have demonstrated increased expression of ZnT-3 and MT-3 as well as the lipid metabolism-related genes ApoE, ApoJ and ACAT-1 in the hippocampus following neonatal seizures, which was inhibited by chronic KD treatment (16). Chronic KD treatment restored the down-regulated lipid membrane peroxidation -associated pathway cPLA2 following neonatal seizure, indicating that cPLA2 may be involved in the zinc/lipid metabolic pathway (17). These results highlight zinc/lipid metabolism pathway as potential targets of KD for the treatment of zinc/lipid peroxidation following developmental seizures. However, which signal plays the key role remains elusive.

Here, we have expanded our previous findings by showing, for the first time, that rats treated with KD (both KD and KOD groups) had a significant downregulation of expression of hippocampal Apoa1, Pdk4 and upregulation of ApoE, ANXN7 and cPLA2 when compared with the non-KD treated ND group (except in the case of ApoE in the KOD group). Meanwhile, ZnTs expressions were not significantly different among the three groups. It has been reported that an 8-week KD may remodel the lipid metabolism profile, thus contributing to influence exercise capacity (33). Zhang et al. showed that a ketogenic diet (KD) improved cellular metabolism and functional recovery after moderate traumatic brain injury in adolescent rats (34). These findings, combined with our present results, reinforce the importance of the lipid metabolism signals as an inducer of alterations in gene expression in the hippocampus and that such changes might contribute to the pathophysiology of the KD in brain disorders.

It is interesting to point out that the expression of cPLA2 in the present study is consistent with the neurobehavioral and cognitive changes in KD-treated rats. The unregulated activation of cPLA2 has been well-documented to be essential for the onset of several neurodegenerative diseases (35–38). Bate C and Williams A recently showed that the αSN-induced activation of cPLA_2_ residing within synapses in cultured neurons was responsible for synaptic injury, which was restored by pretreatment with the inhibitor of cPLA_2_ (39). Liu et al. found that spinal cord injury (SCI) significantly increased cPLA2 expression and activation. Remarkably, blocking cPLA2 improved motor deficits after spinal cord injury and ameliorated cell loss and tissue damage (40). There are few studies on brain cPLA2 expression after epilepsy attack. Sandhya TL found that kainic acid injection caused an increase in cPLA2 immunoreactivity in hippocampal neurons of astrocytes at different time points in acute and long-term, suggesting that cPLA2 may be involved in neurodegeneration (41). We recently showed that neonatal seizures induced a long-term lower amount of cPLA2 in hippocampus, which was in parallel with hippocampal mossy fiber sprouting and cognitive deficits. Furthermore, chronic KD treatment effectively restored these molecular, neuropathological, and cognitive changes (17). It should be pointed out that in the study there were no significant changes in protein level of cPLA2 in hippocampus between the two control groups (ND vs. KD). However, in our present results, both the mRNA and protein levels of cPLA2 in the KOD and KD rats were significantly upregulated compared with the ND group. The main reason for this discrepancy may be due to total intake of KD. In previous study, the KD started on P21 for 3 consecutive weeks; in present study, however, the KD started on P28 for 30 consecutive days. Therefore, the total amount of KD intake in this study is significantly higher than the former. Taken together, it is reasonable to speculate that cPLA2 may be an attractive therapeutic target for KD to ameliorate long-term brain injury following developmental seizures.

## Conclusion

This study demonstrates that early-life KD can provoke minor neurobehavioral effetcs in particular a delay in negative geotaxis reflex and an increase in grooming activity. Likewise, KD treatment induced significantly downregulated mRNA levels of Apoa1, Pdk4, and upregulated expression of ApoE, ANXN7, and cPLA2 when compared to control. The upregulation of cPLA_2_ was also confirmed on the protein level. The results suggest that hippocampal lipid metabolism signaling pathway, especially cPLA2, may be the target of the protective effect of KD on long-term brain injury after developmental seizures and deserves further study.

## Author Contributions

HN was the designer and dissertation writer of this study. YL, DW, YS, and DZ were the operators of this experiment and were responsible for the statistical analysis of data.

### Conflict of Interest Statement

The authors declare that the research was conducted in the absence of any commercial or financial relationships that could be construed as a potential conflict of interest.

## Supplementary Material

Supplementary Figure 1 The animals' weight at P49.
