# Supplementary figures and images for: Neuro-Behavioral Status and the Hippocampal Expression of Metabolic Associated Genes in Wild-Type Rat Following a Ketogenic Diet
Source: Front Neurol. 2019 Feb 5;10:65. doi: 10.3389/fneur.2019.00065 (PMC6370680; doi:10.3389/fneur.2019.00065)

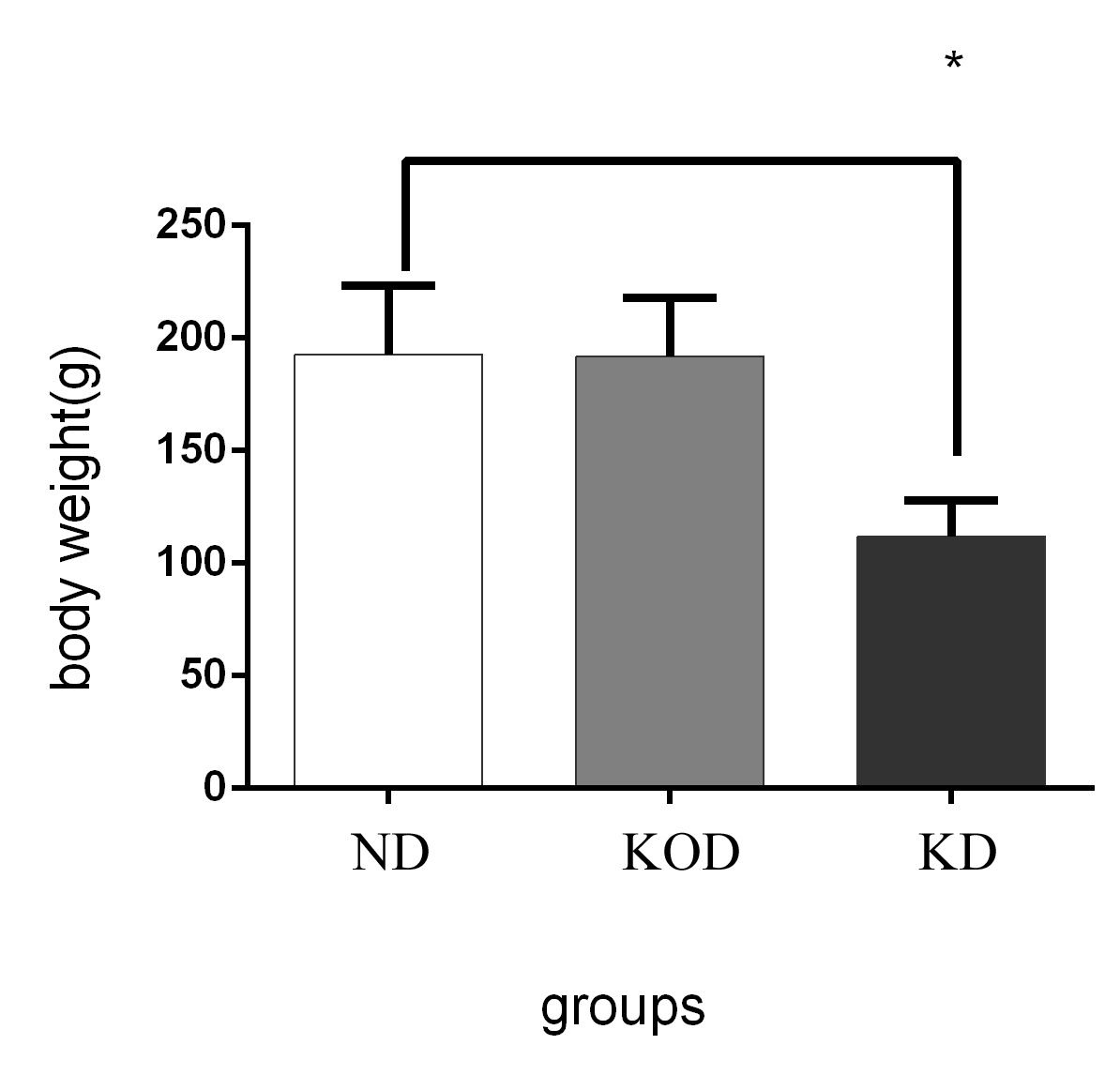

Supplement: Supplementary Figure 1 — The animals' weight at P49. [file Image_1.jpg]
